# Supplementary material for: The ubiquitin-conjugating enzyme UBE2K determines neurogenic potential through histone H3 in human embryonic stem cells
Source: Commun Biol. 2020 May 25;3:262. doi: 10.1038/s42003-020-0984-3 (PMC7248108; doi:10.1038/s42003-020-0984-3)
Supplement: Supplementary file 1 — Supplementary Information [file 42003_2020_984_MOESM1_ESM.pdf]

Supplementary Figures

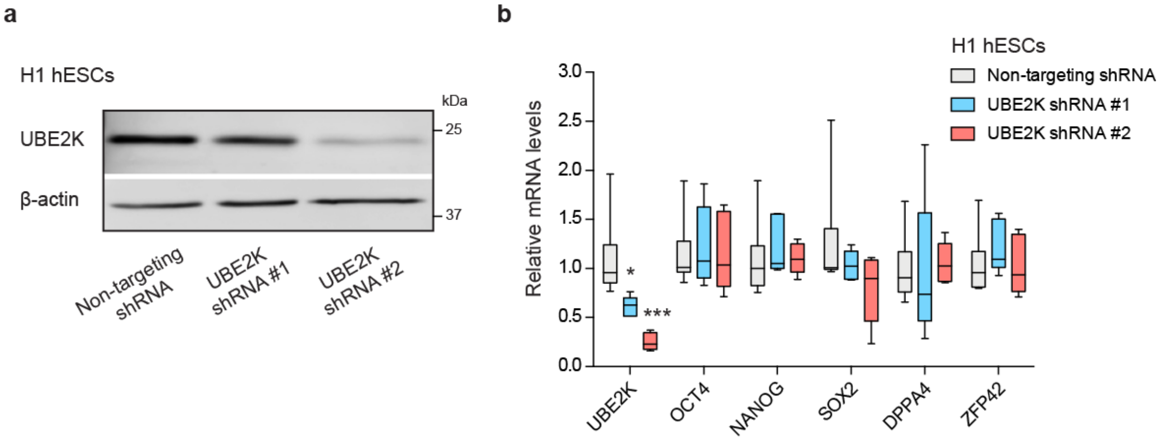

Supplementary Figure 1. Loss of UBE2K does not impair the expression of pluripotency markers in H1 hESCs. a, Western blot analysis of H1 hESCs with antibody to UBE2K. β-actin is the loading control. Images are representative of two independent experiments. **b,** qPCR analysis of UBE2K and pluripotency markers in H1 hESCs. Graph (relative expression to non-targeting (NT) shRNA control hESCs) represents the mean ± s.e.m. (NT shRNA n= 6, UBE2K shRNA #1 n= 5, UBE2K shRNA #2 n= 6 independent experiments). All the statistical comparisons were made by two-tailed Student's t-test for unpaired samples. P-value: *(P<0.05), ***(P<0.001).

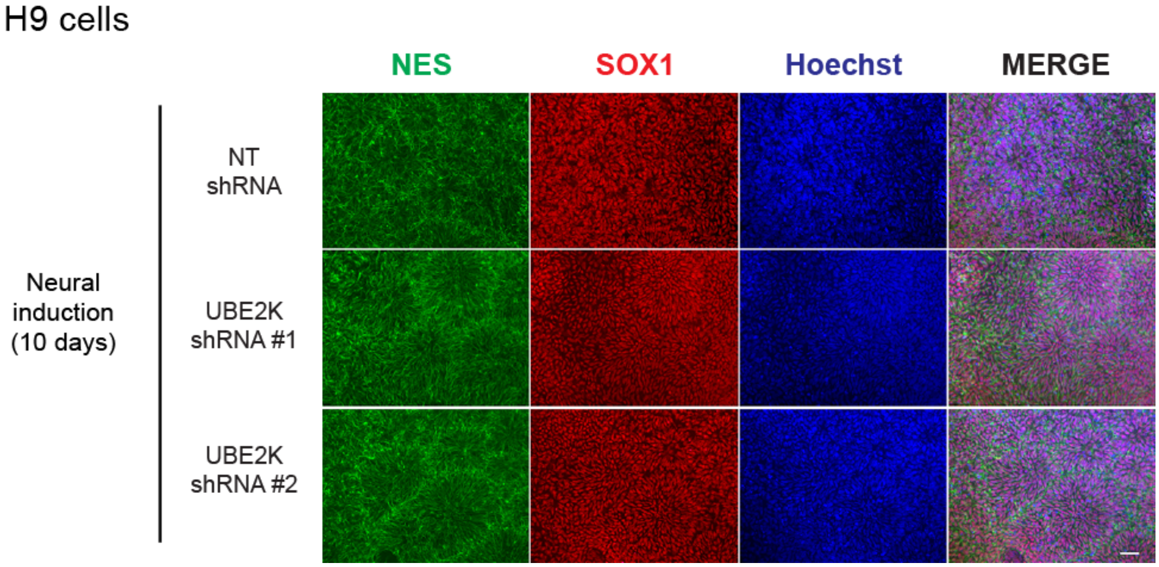

Supplementary Figure 2. Loss of UBE2K does not impair the induction of Nestin and SOX1 during the early stages of differentiation into NPCs. After 10 days of neural induction, H9 cells were assessed by immunofluorescence with NES and SOX1 staining. Hoechst staining was used as a marker of nuclei. Scale bar represents 40 µm. The images are representative of two independent experiments.

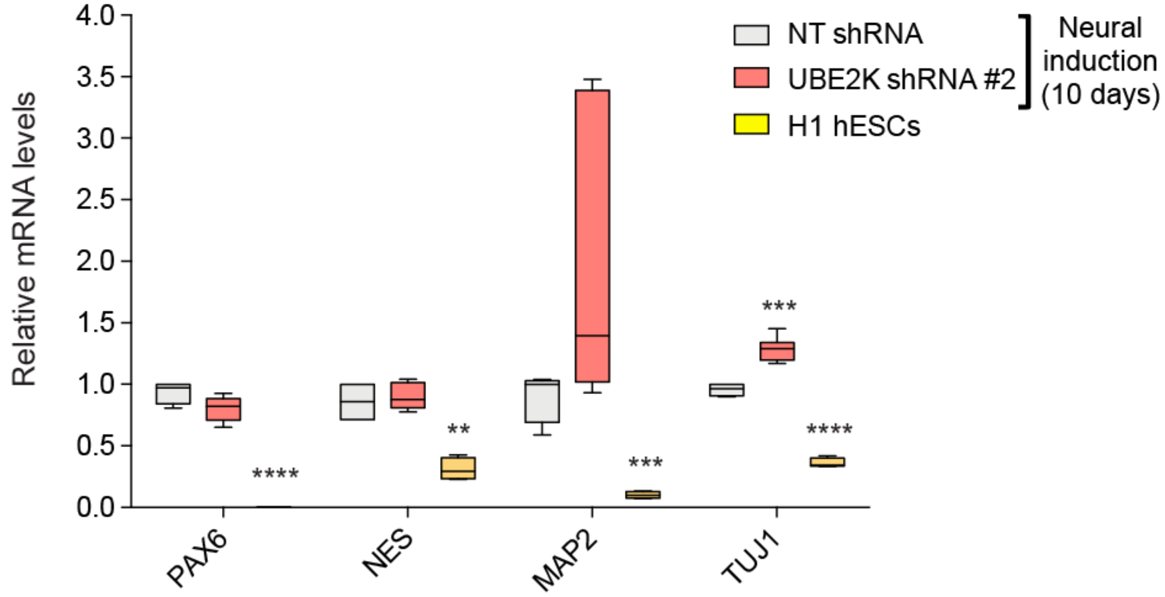

Supplementary Figure 3. Loss of UBE2K does not impair the early stages of neural induction in H1 hESCs. qPCR analysis after 10 days of neural induction of H1 hESCs. Graph (relative expression to NT shRNA cells) represents the mean ± s.e.m. (NT shRNA n= 4, UBEK shRNA #2 n=6, hESCs n= 4 independent experiments). All the statistical comparisons were made by two-tailed Student's t-test for unpaired samples. P-value: ** (P<0.01), *** (P<0.001),****(P<0.0001).

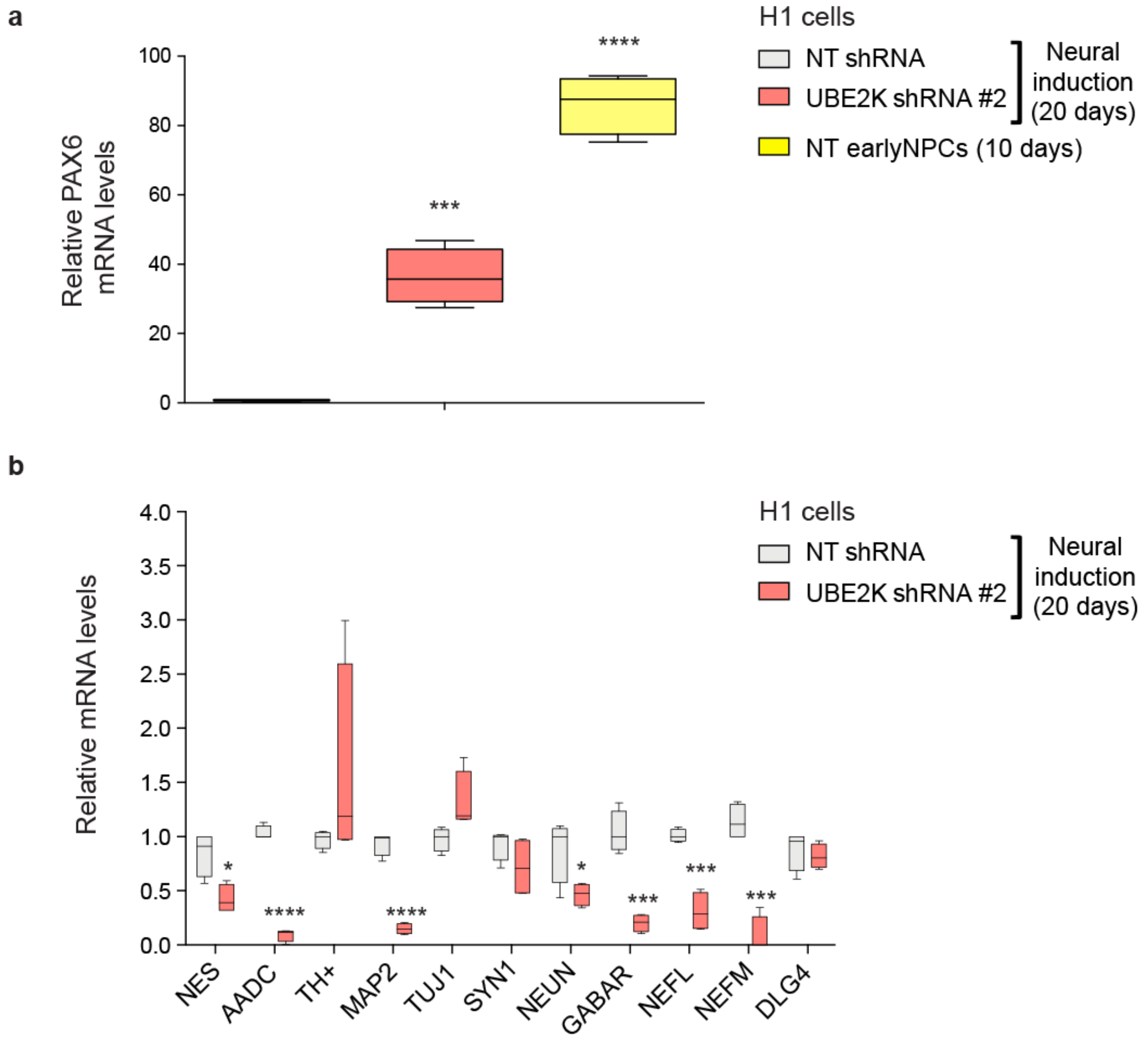

Supplementary Figure 4. Loss of UBE2K in H1 hESCs impairs their differentiation into mature NPCs. a, qPCR analysis of PAX6 transcripts in H1 cells after 20 days on neural induction treatment. Graph (relative expression to NT shRNA cells) represents the mean ± s.e.m. of four independent experiments. **b,** qPCR analysis of distinct neural and neuronal markers after 20 days of neural induction of H1 hESCs. Graph (relative expression to NT shRNA cells) represents the mean ± s.e.m. of four independent experiments. All the statistical comparisons were made by two-tailed Student's t-test for unpaired samples. P-value: *(P<0.05), ***(P<0.001), ****(P<0.0001).

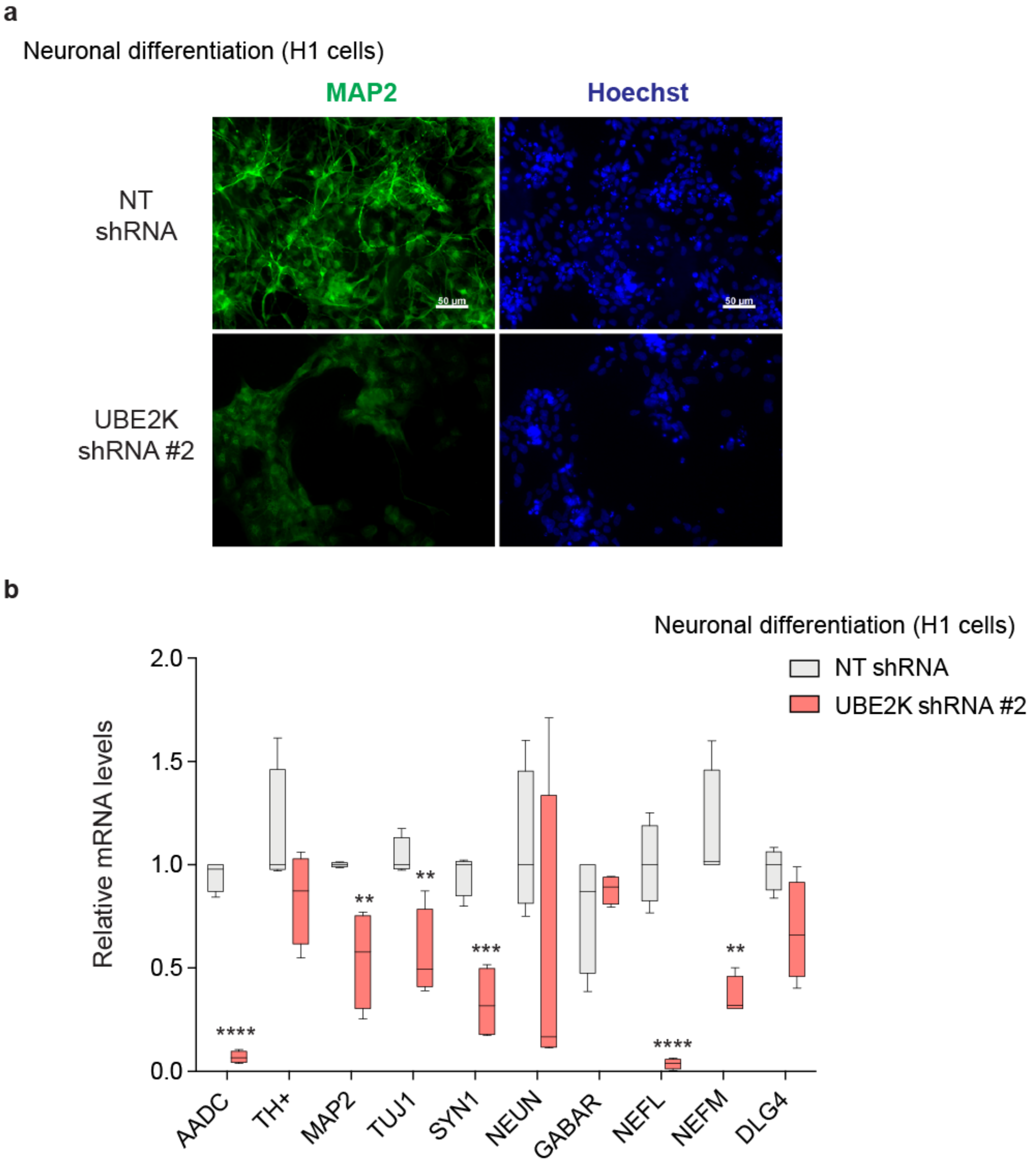

Supplementary Figure 5. NPCs derived from UBE2K shRNA H1 hESCs exhibit impaired neuronal differentiation. a, After pan-neuronal differentiation (H1 line), cells were assessed by immunofluorescence with MAP2 and Hoechst staining. Scale bar represents 50 µm. Images are representative of two independent experiments. **b,** qPCR analysis after pan-neuronal differentiation of H1 hESCs. Graph (relative expression to NT shRNA H1 cells) represents the mean ± s.e.m. of four independent experiments All the statistical comparisons were made by two-tailed Student's t-test for unpaired samples. P-value: ** ($P<0.01$), ***($P<0.001$), ****($P<0.0001$).

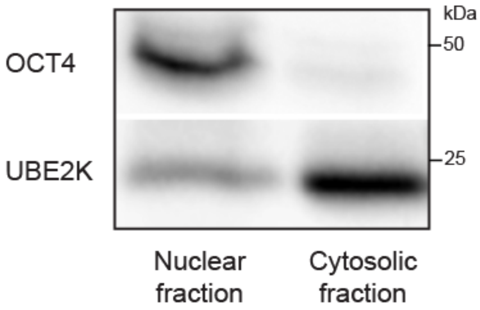

Supplementary Figure 6. UBE2K localizes in both the nucleus and the cytoplasm. Western blot analysis of nuclear and cytosolic fractions from H9 hESCs with antibodies to the transcription factor OCT4 and UBE2K.

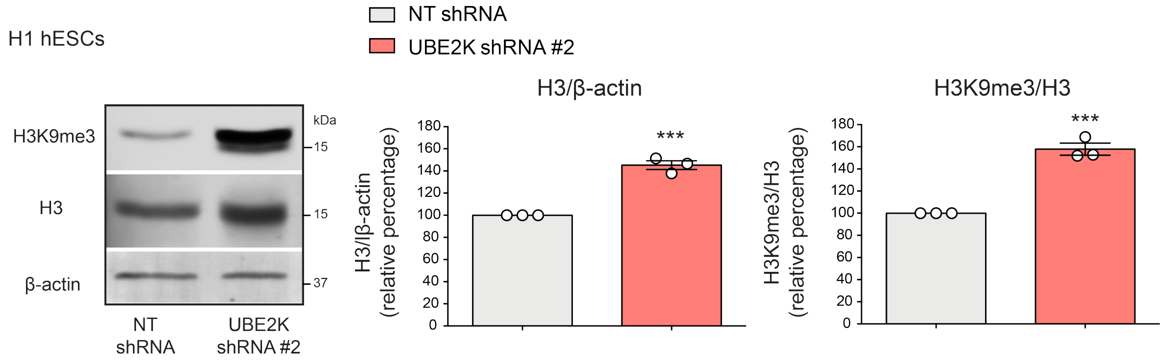

Supplementary Figure 7. Loss of UBE2K up-regulates total histone H3 levels and trimethylation of H3K9 in H1 hESCs. Western blot analysis of H1 hESCs with antibodies to H3K9me3 and total H3. Graphs represent the relative percentage values of H3/β-actin and H3K9me3/H3 to NT shRNA. All the statistical comparisons were made by two-tailed Student's t-test for unpaired samples (mean ± s.e.m., three independent experiments). P-value: ***(P<0.001).

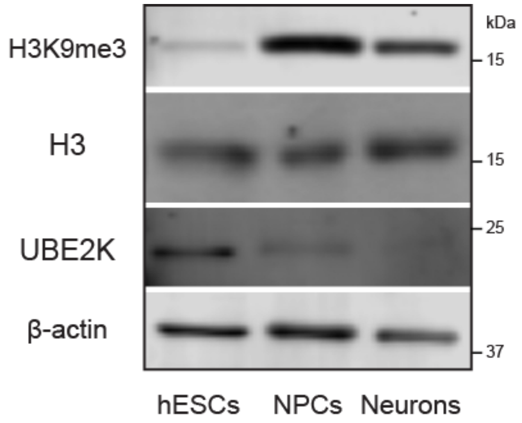

Supplementary Figure 8. Histone H3 and H3K9me3 levels during differentiation. Western blot analysis of H9 cells with antibodies to H3K9me3, total H3, UBE2K and β-actin. The images are representative of three independent experiments.

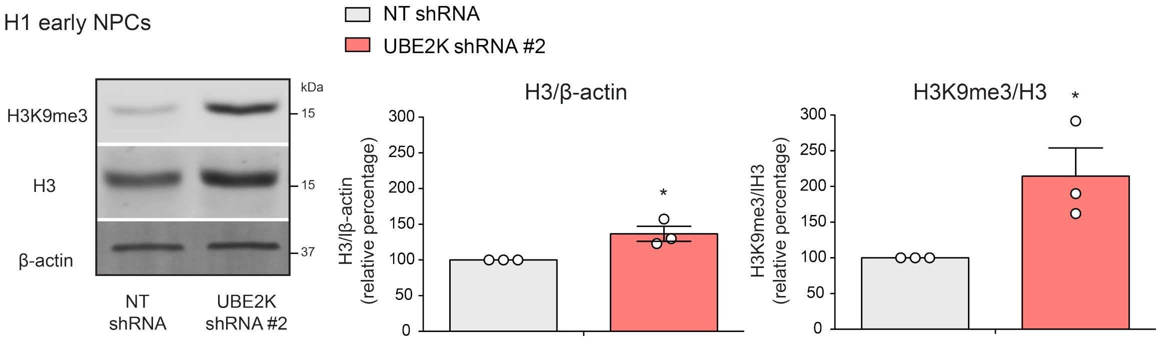

Supplementary Figure 9. Early NPCs derived from UBE2K shRNA H1 hESCs retain abnormal high levels of total H3 and H3K9me3/H3 ratio. After 10 days of neural induction of UBE2K shRNA H1 hESCs, early NPCs were analysed by western blot with antibodies to H3K9me3 and total H3. Graphs represent the H3/β-actin and H3K9me3/H3 relative percentage values to NT shRNA H1 NPCs. All the statistical comparisons were made by two-tailed Student's t-test for unpaired samples (mean ± s.e.m., three independent experiments). P-value: *($P<0.05$).

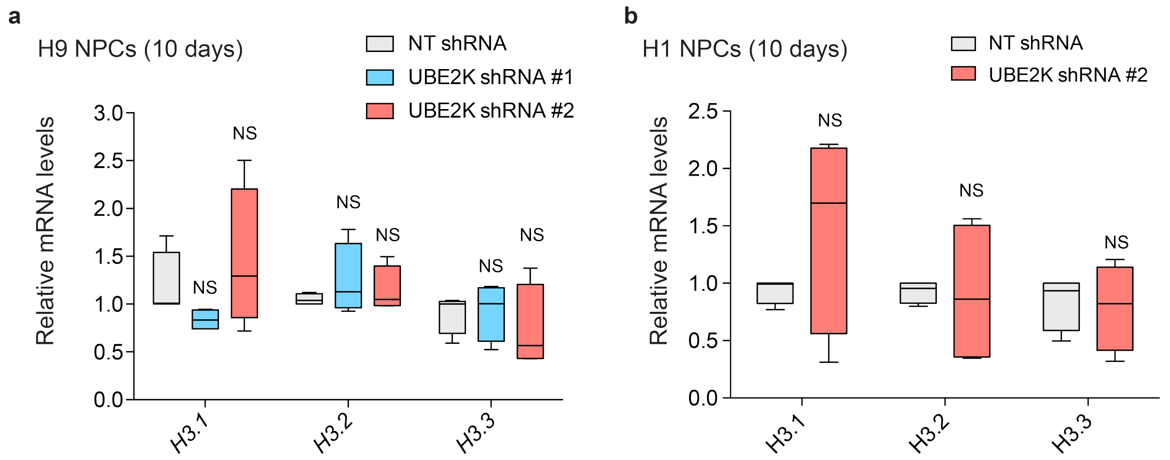

Supplementary Figure 10. Early NPCs derived from UBE2K shRNA hESCs do not exhibit significant changes in histone H3 transcripts. a, After 10 days on neural induction treatment of H9 UBE2K KD hESCs, we analyzed the mRNA levels of histone H3 variants in early NPCs. Graph (relative expression to NT shRNA control NPCs) represents the mean ± s.e.m. of four independent experiments. **b,** qPCR analysis of histone H3 variants in early H1 NPCs (10 days on neural induction). Graph (relative expression to NT shRNA control NPCs) represents the mean ± s.e.m. of four independent experiments. All the statistical comparisons were made by two-tailed Student's t-test for unpaired samples. NS= not significant (P>0.05).

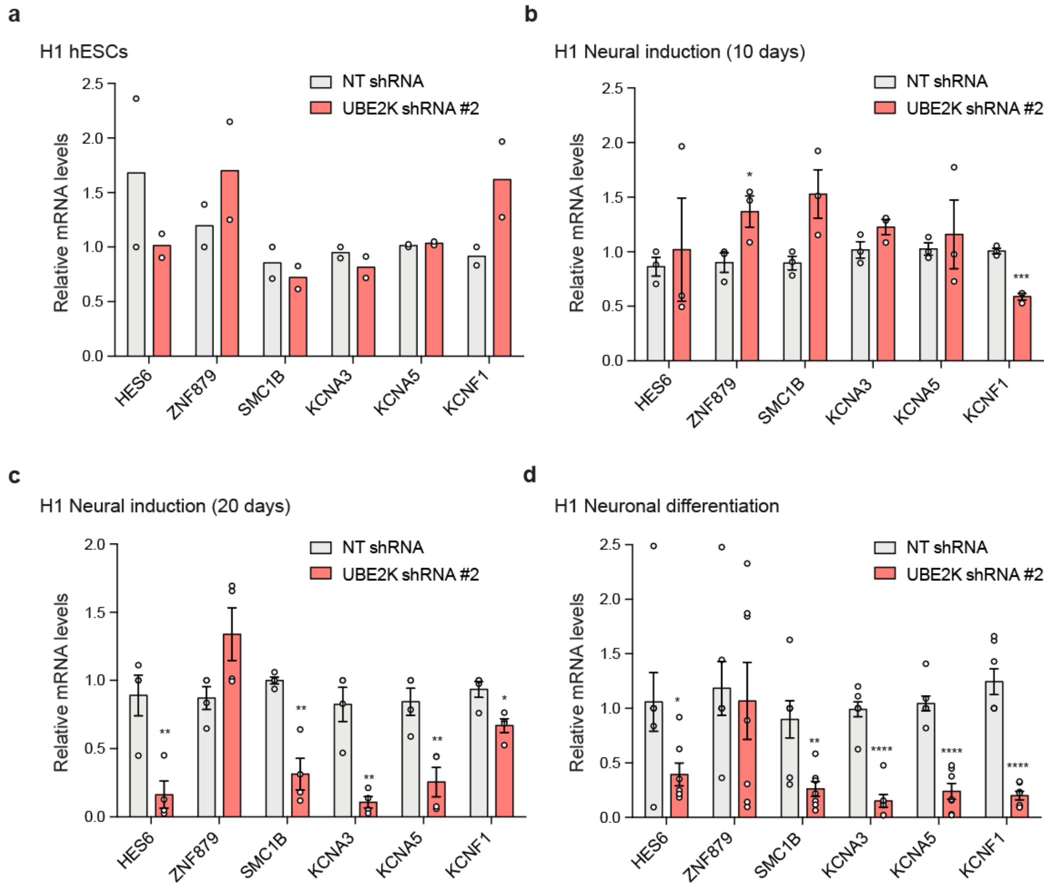

Supplementary Figure 11. Loss of UBE2K in H1 hESCs diminishes the induction of neuronal genes during differentiation. a, qPCR analysis of H1 hESCs. Graph (relative expression to NT shRNA) represents the mean ± s.e.m. of two independent experiments. **b,** qPCR analysis after 10 days of neural induction of H1 hESCs. Graph (relative expression to NT shRNA) represents the mean ± s.e.m. of three independent experiments. **c,** qPCR analysis after 20 days of neural induction of H1 hESCs. Graph (relative expression to NT shRNA) represents the mean ± s.e.m. of four independent experiments. **d,** qPCR analysis after neuronal differentiation (H1 line). Graph (relative expression to NT shRNA) represents the mean ± s.e.m. of seven independent experiments. Statistical comparisons were made by two-tailed Student's t-test for unpaired samples. P-value: *(P<0.05), **(P<0.01), ***(P <0.001), ****(P<0.0001).

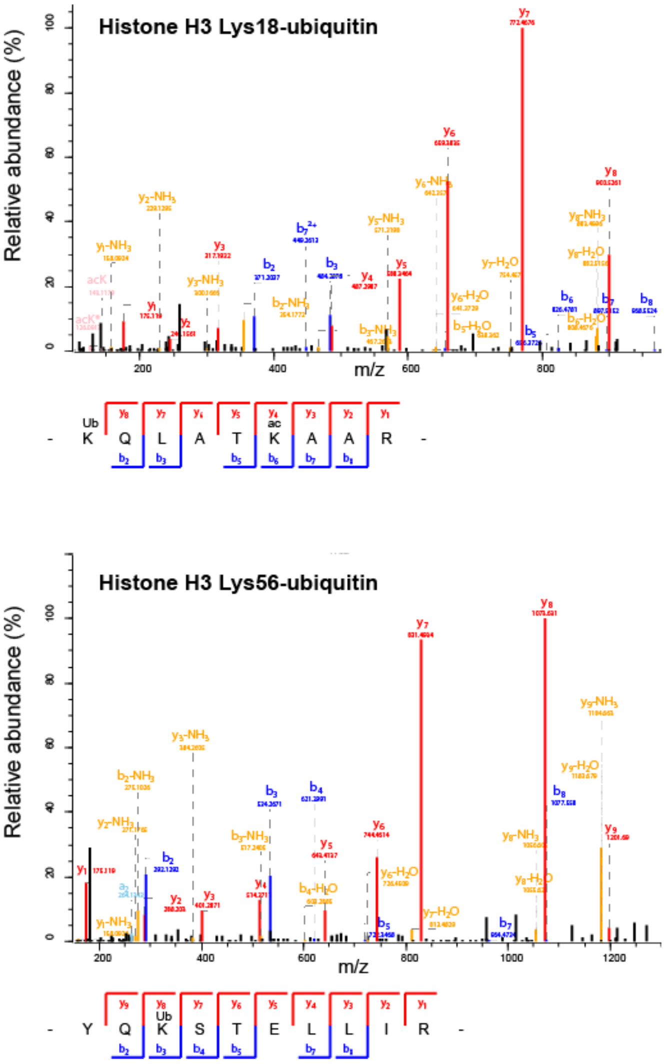

Supplementary Figure 12. Histone H3 exhibits ubiquitination events at Lys18 and Lys56. High-resolution of higher collisional dissociation fragmentation spectrum of histone H3 peptides modified by ubiquitin on Lys18 and Lys56 (n= 4). Peptide sequence and fragment ions assignment are shown below the spectrum.

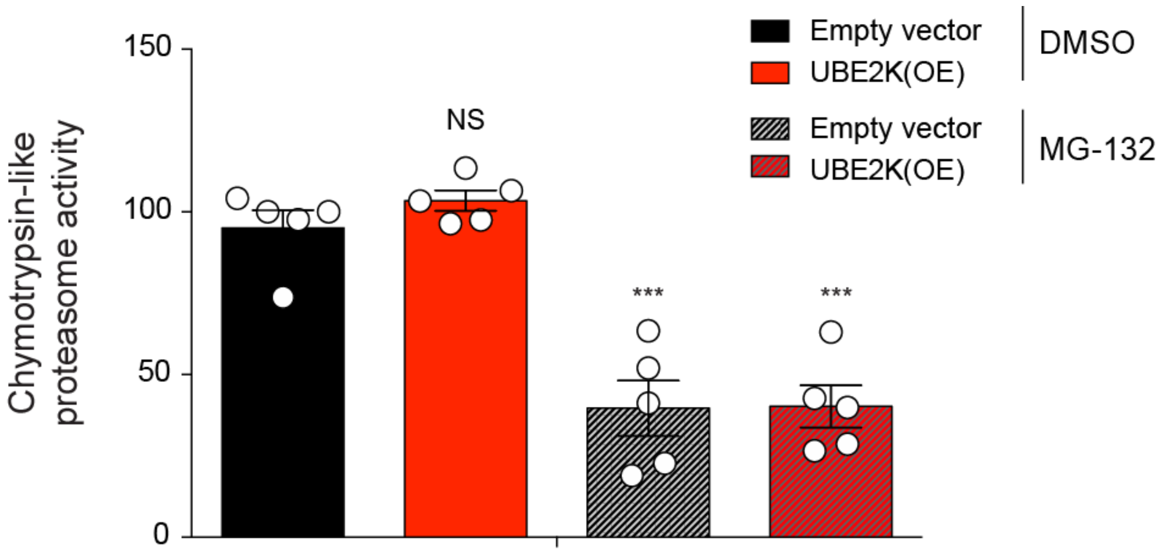

Supplementary Figure 13. Ectopic expression of UBE2K does not affect proteasome activity. Chymotrypsin-like proteasome activity in HEK293 cells. Empty vector and UBE2K overexpressing (OE)-cells have similar proteasome activity. The treatment with 5 μM MG-132 for 16 h induces a similar decrease in proteasome activity in both empty vector and UBE2K(OE) cells. The graph represents the relative percentage to Empty vector + DMSO control cells (mean ± s.e.m. of five independent experiments). Statistical comparisons were made by two-tailed Student's t-test for unpaired samples. P-value: ***(P<0.001). NS= not significant.

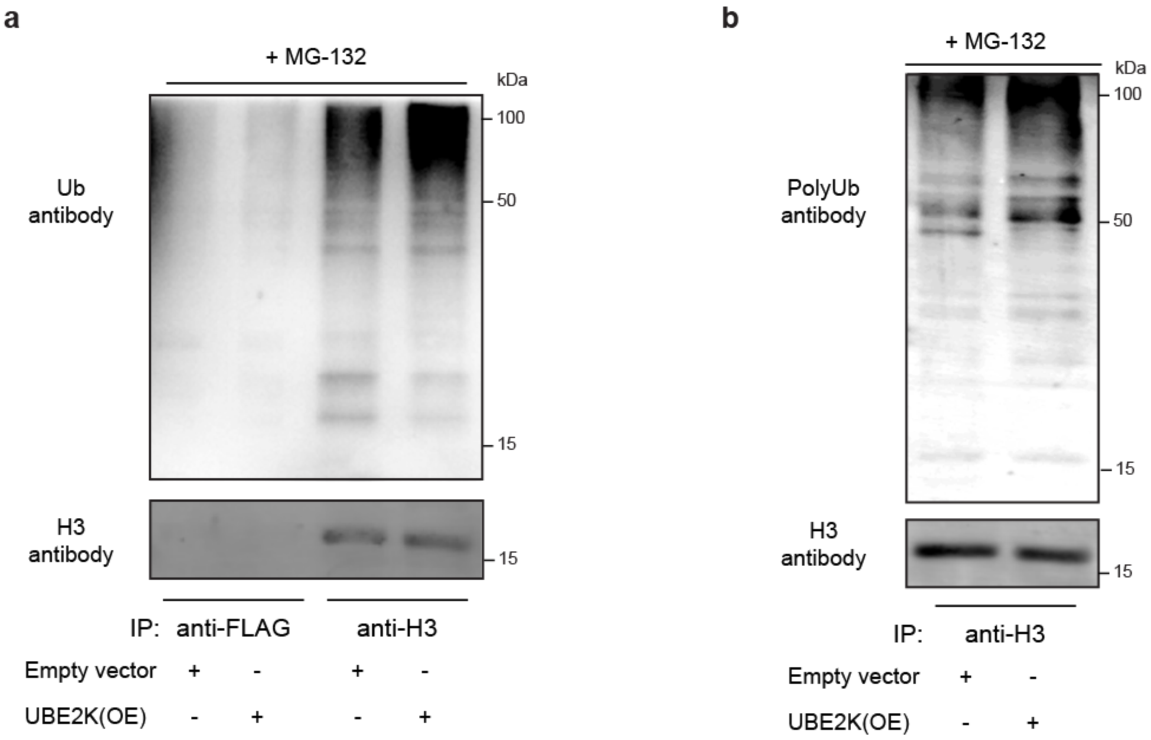

Supplementary Figure 14. Immunoprecipitation of histone H3 followed by western blot with anti-ubiquitin and poly-ubiquitin antibodies. a, Immunoprecipitation (IP) with anti-histone H3 and anti-FLAG antibodies in HEK293 cells. IP was followed by western blot with antibodies to H3 and ubiquitin to detect immunoprecipitated total H3 protein and ubiquitinated H3, respectively. The images are representative of two independent experiments. **b,** IP with anti-histone H3 in HEK293 cells. Immunoprecipitation was followed by western blot with antibodies to H3 and polyubiquitinated proteins (polyUb) to detect immunoprecipitated total H3 protein and polyUb-H3, respectively. The images are representative of two independent experiments. Prior immunoprecipitation, cells were treated with proteasome inhibitor (5 µM MG-132, 16 h) to block the degradation of H3 induced by UBE2K(OE) so we could immunoprecipitate similar H3 amounts for direct comparison of polyubiquitination among the distinct conditions.

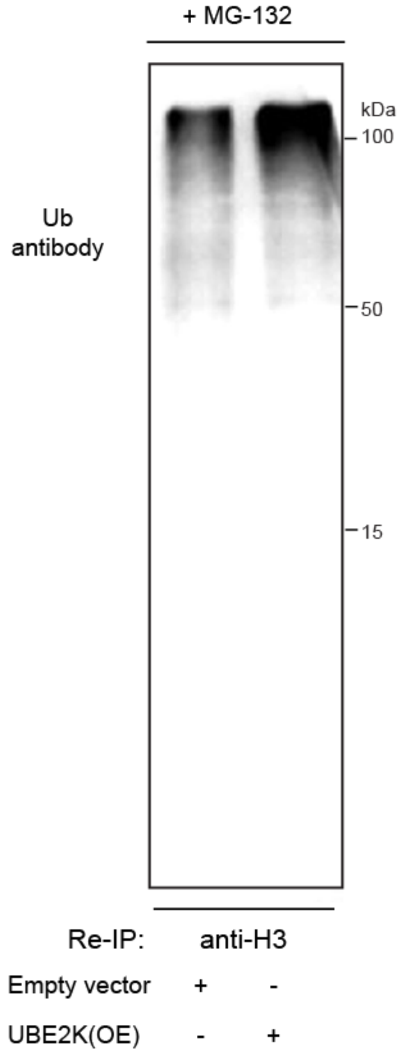

Supplementary Figure 15. Re-immunoprecipitation of histone H3 followed by western blot with anti-ubiquitin antibody. After the first immunoprecipitation with anti-histone H3 antibody in HEK293 cells, we performed a re-immunoprecipitation (Re-IP) with H3 antibody to exclude potential contaminating proteins. Re-IP was followed by western blot with antibody against ubiquitin to detect ubiquitinated H3. The samples presented in this figure correspond to the same experiments showed in figure 9i. Please see Figure 9i for levels of immunoprecipitated total H3 protein and polyUb-H3. The images are representative of two independent experiments.

Supplementary Table

	NPCs		Neurons	
	T-test difference	q-value	T-test difference	q-value
**UBE2C**	-1.54	<0.0001	-2.47	<0.0001
**UBE2G1**	-1.15	0.0005	-1.43	0.0001
**UBE2K**	-1.04	0.0015	-1.03	0.0017
**UBE2O**	-0.75	0.0241	-1.39	0.0002
**UBE2Z**	-0.49	0.0164	-0.08	NS
**UBE2S**	-0.98	NS	-1.71	0.0012
**UBE2V1**	-0.01	NS	0.44	NS
**UBE2V2**	0.06	NS	0.40	0.0090
**UBE2N**	0.09	NS	0.04	NS
**UBE2R2**	0.14	NS	0.01	NS
**UBE2M**	0.20	NS	0.39	NS
**UBE2Q1/UBE2Q2**	0.28	NS	0.81	NS
**UBE2I**	0.83	0.0007	0.15	NS
**UBE2L3**	1.26	<0.0001	0.76	<0.0001

Supplementary Table 1. Quantitative proteomic analysis of E2 enzymes comparing hESCs with their NPC and neuronal counterparts. UBE2K decreases during neural differentiation of hESCs. We used limma's moderated t-test to contrast hESCs (n= 9) versus NPCs (n= 5) and neurons (n= 6). Relative abundance differences are calculated from the log2 of label-free quantification (LFQ) values (LFQ NPCs/hESCs and LFQ Neurons/hESCs). Adjusted p-value (q-value) of <0.05 is considered significant. NS= non-significant.
