# Supplementary material for: The ubiquitin-conjugating enzyme UBE2K determines neurogenic potential through histone H3 in human embryonic stem cells
Source: Commun Biol. 2020 May 25;3:262. doi: 10.1038/s42003-020-0984-3 (PMC7248108; doi:10.1038/s42003-020-0984-3)
Supplement: Supplementary file 2 — Supplementary Information [file 42003_2020_984_MOESM2_ESM.pdf]

Supplementary Data 1. Transcriptomic data of UBE2K KD (shRNA #1) H9 hESCs compared with non-targeting (NT) shRNA H9 hESCs (n=3 biologically independent samples; p-value <0.05, Fold change >2 was considered significant). This file also presents GOBP analysis of down-regulated and up-regulated transcripts in UBE2K shRNA hESCs.

Supplementary Data 2. Proteomic analysis of UBE2K shRNA hESCs compared with NT shRNA control hESCs (n=5 biologically independent samples, False Discovery Rate (FDR)<0.05 was considered significant).

Supplementary Data 3. List of proteins significantly down-regulated in both UBE2K knockdown hESC lines (shRNA #1 and shRNA #2). Means are calculated from the log2 of label-free quantitative (LFQ) values (LFQ UBE2K shRNA H9 hESCs/Non-targeting shRNA H9 hESCs). Statistical comparisons were made by Student's t-test (n=5 biologically independent samples, FDR< 0.05 was considered significant). This file also presents GOBP analysis of down-regulated proteins in UBE2K shRNA hESCs.

Supplementary Data 4. List of proteins significantly up-regulated in both UBE2K knockdown hESC lines (shRNA #1 and shRNA #2). Means are calculated from the log2 of label-free quantitative (LFQ) values (LFQ UBE2K shRNA H9 hESCs/Non-targeting shRNA H9 hESCs). Statistical comparisons were made by Student's t-test (n=5 biologically independent samples, FDR< 0.05 was considered significant). This file also presents GOBP analysis of up-regulated proteins in UBE2K shRNA hESCs.

Supplementary Data 5. ChIP-seq with antibody to H3K9me3 comparing UBE2K shRNA (#1 + #2) hESCs with non-targeting shRNA hESCs (2 independent experiments). Fold-change >1.5 enrichment + p-value < 0.05 was considered significant.

Supplementary Data 6. List of primers used for qPCR analysis.

Supplementary Data 7. Uncropped images of the western blots presented in the main and supplementary figures.

Supplementary Data 8. All source data underlying the graphs presented in the main figures.
