# Supplementary material for: The ubiquitin-conjugating enzyme UBE2K determines neurogenic potential through histone H3 in human embryonic stem cells
Source: Commun Biol. 2020 May 25;3:262. doi: 10.1038/s42003-020-0984-3 (PMC7248108; doi:10.1038/s42003-020-0984-3)

Supplementary Data 7. Uncropped images of the western blots presented in the main and supplementary figures.

Fig. 1a

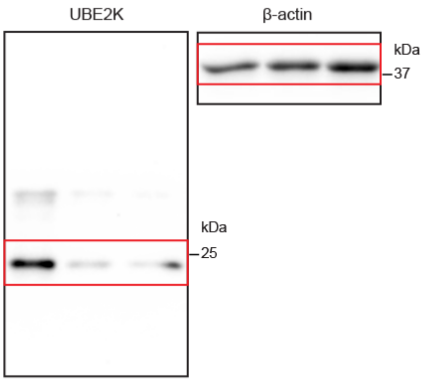

Fig. 1c

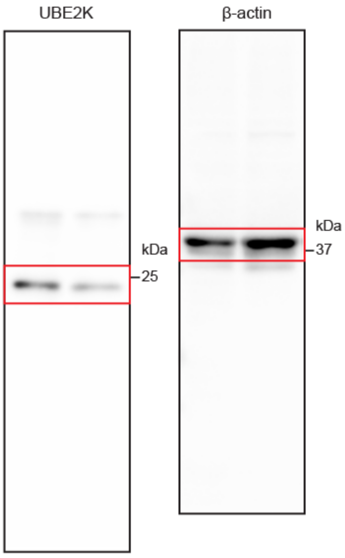

Fig. 1d

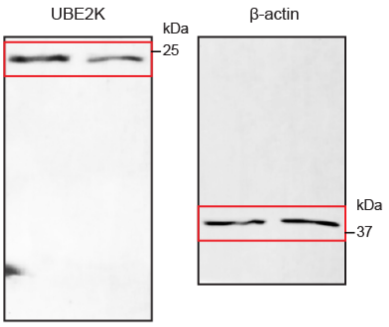

Fig. 1e

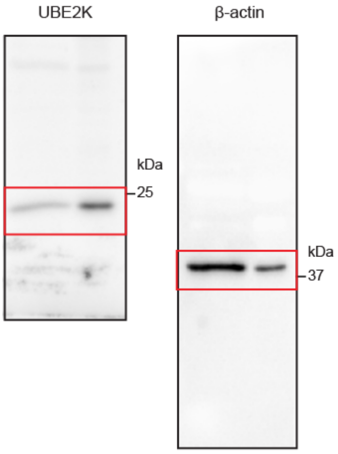

Fig. 2d

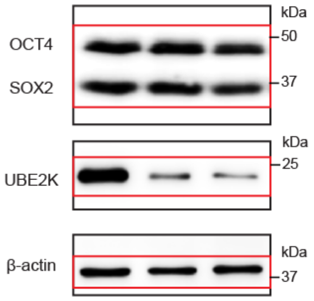

Supplementary Fig. 1a

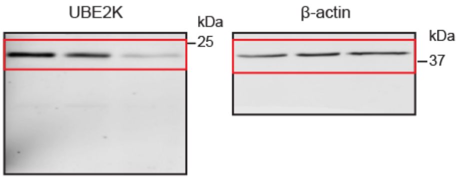

Fig. 3b

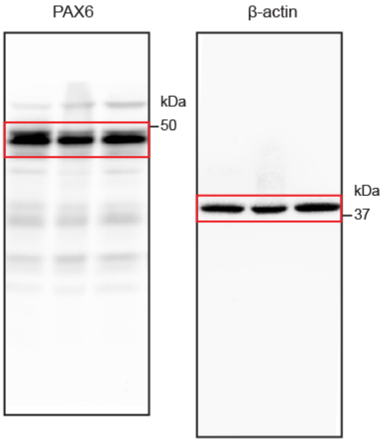

Fig. 3E

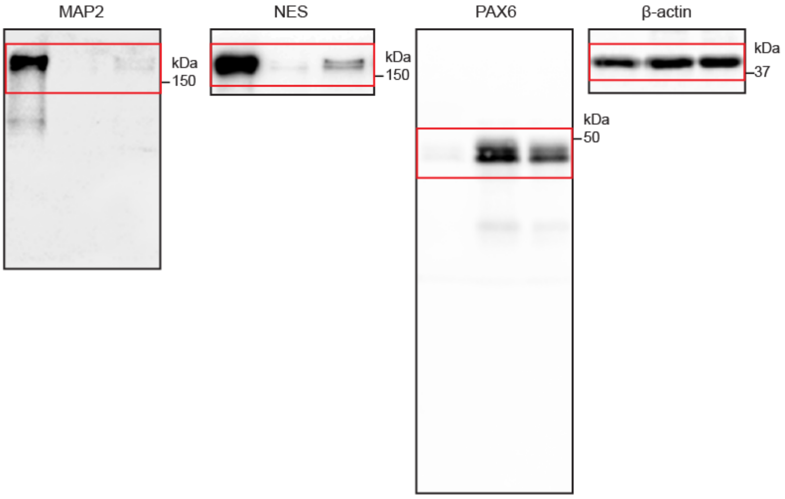

Supplementary Fig. 6

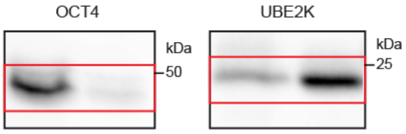

Fig. 4a

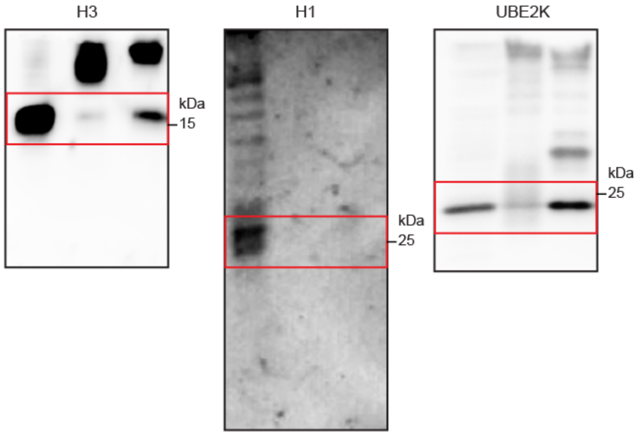

Fig. 4b

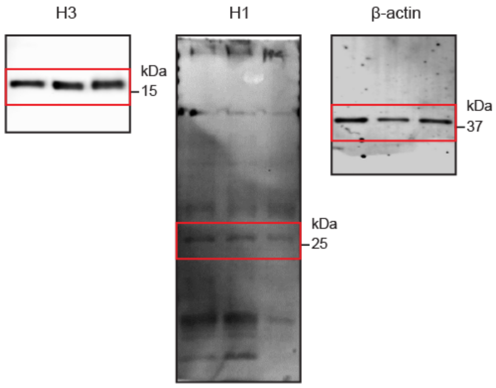

Fig. 4d

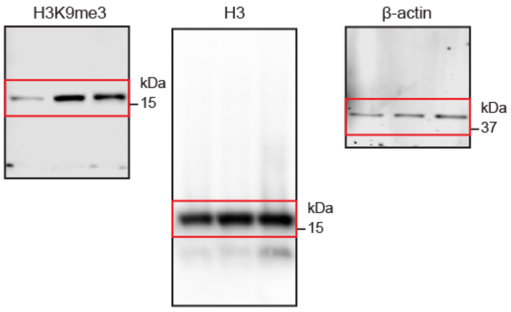

Supplementary Fig. 7

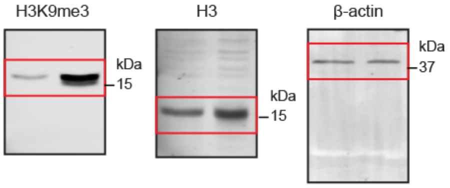

Fig. 5a

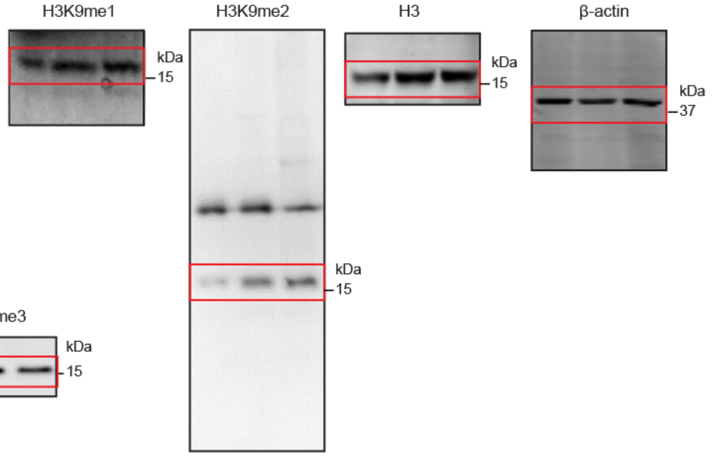

Fig. 5b

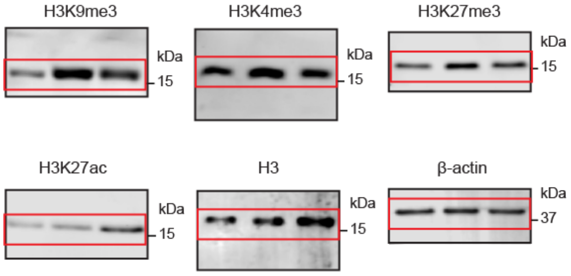

Fig. 5c

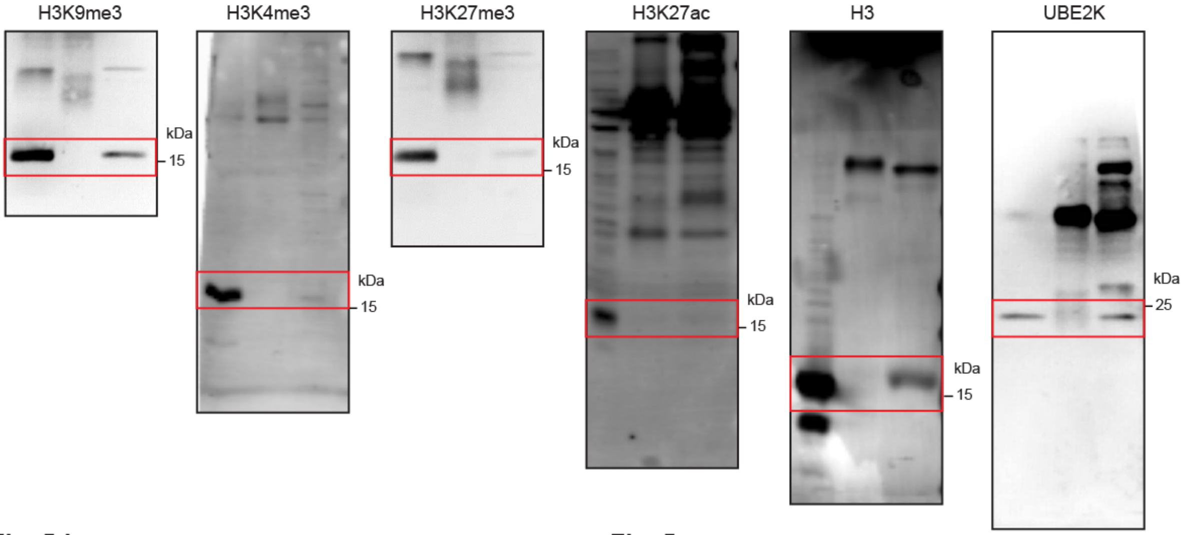

Fig. 5d

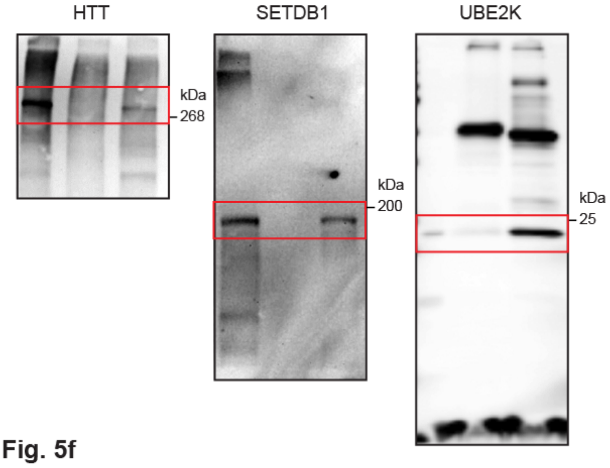

Fig. 5e

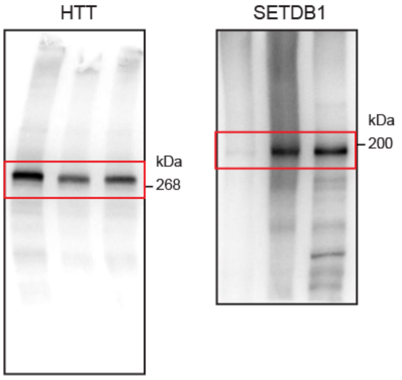

Fig. 5f

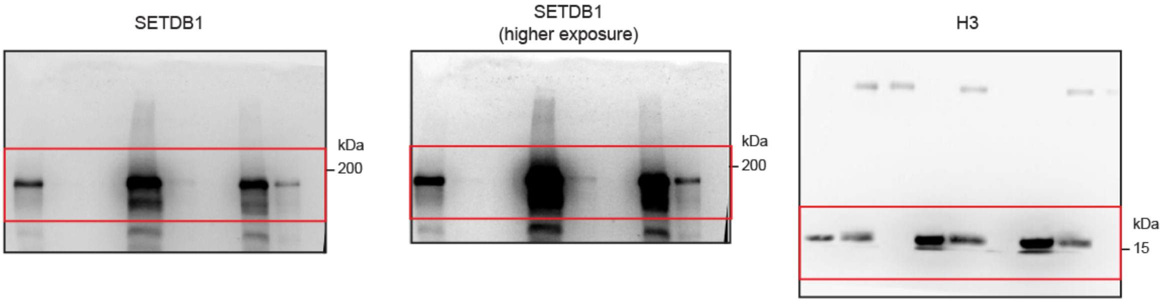

Supplementary Fig. 8

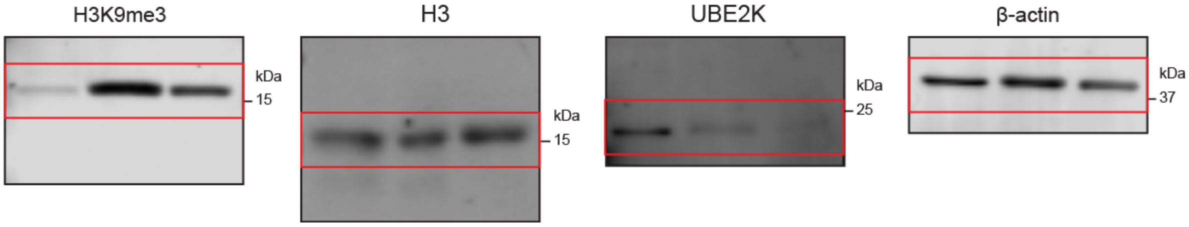

Fig. 6a

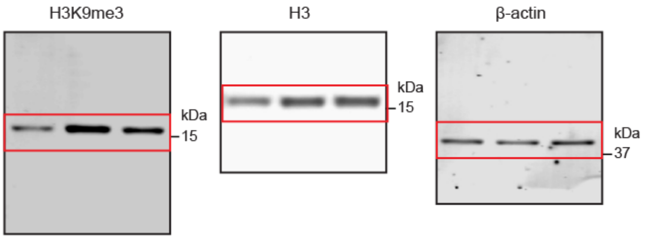

Supplementary Fig. 9

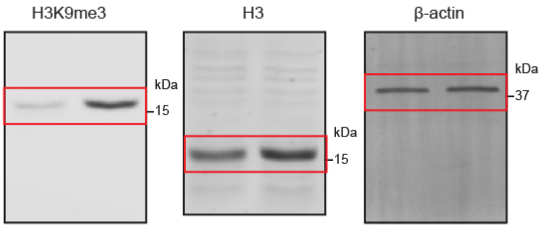

Fig. 8a

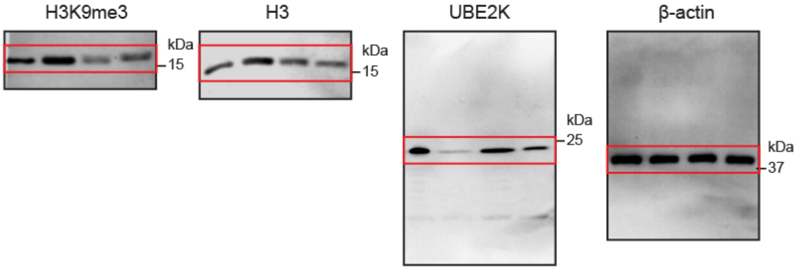

Fig. 9a

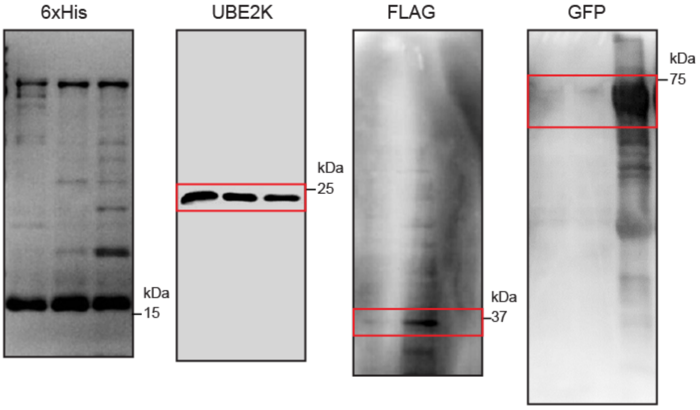

Fig. 9b

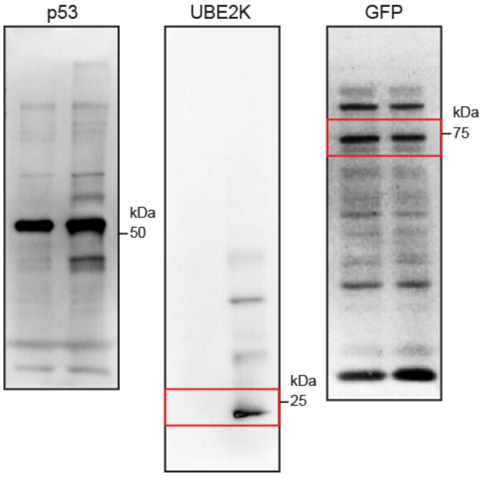

Fig. 9c

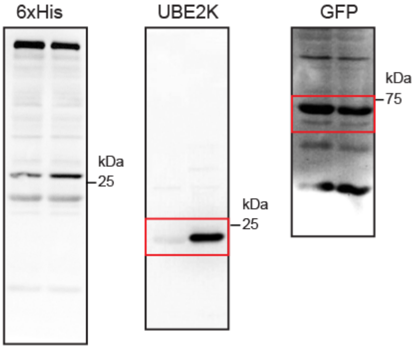

Fig. 9d

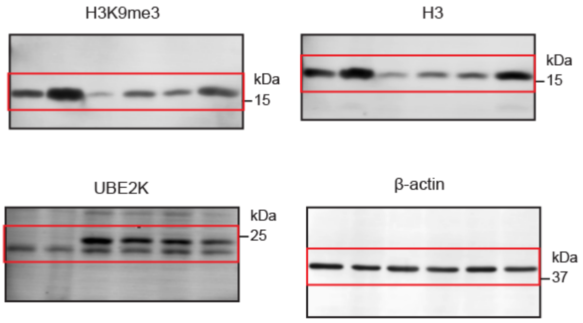

Fig. 9g

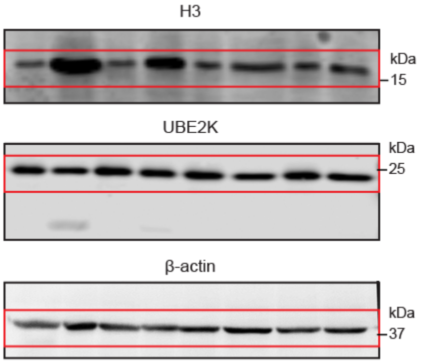

Fig. 9h

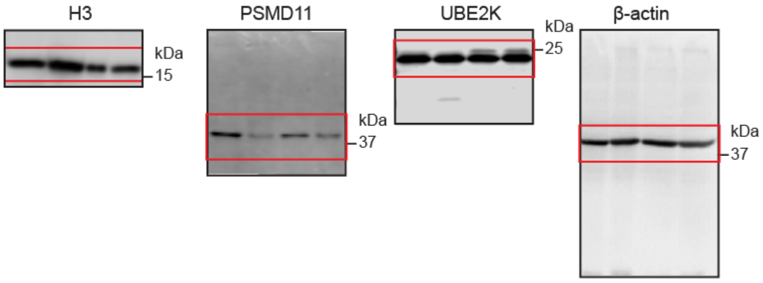

Fig. 9i

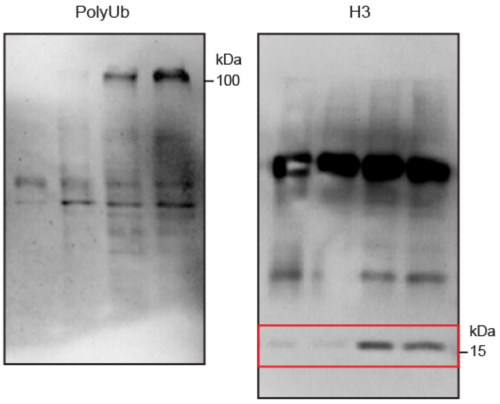

Supplementary Fig. 14a

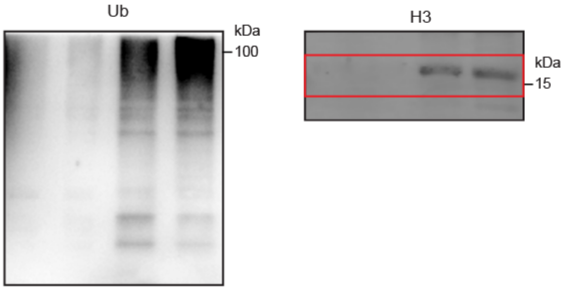

Supplementary Fig. 14b

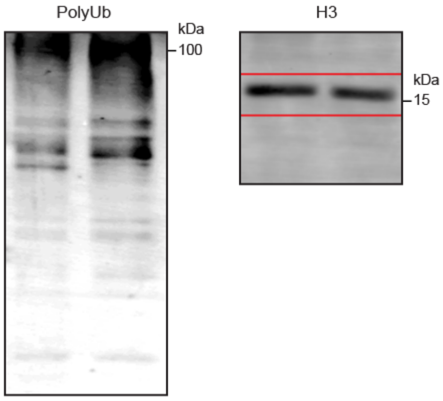

Supplementary Fig. 15

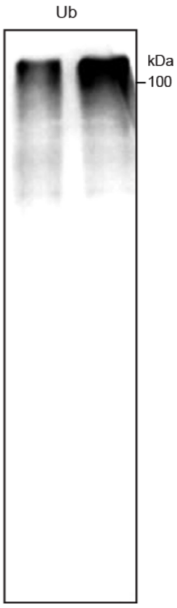

Fig. 10a

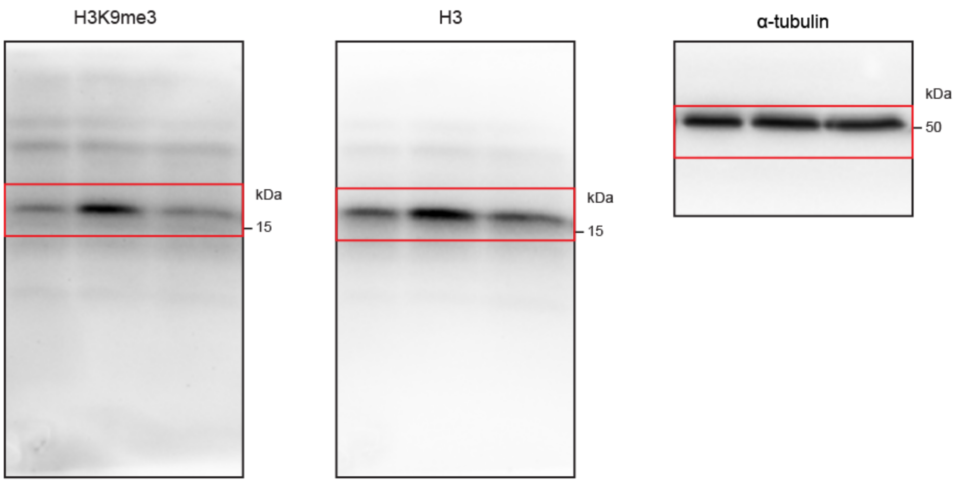

Fig. 10b

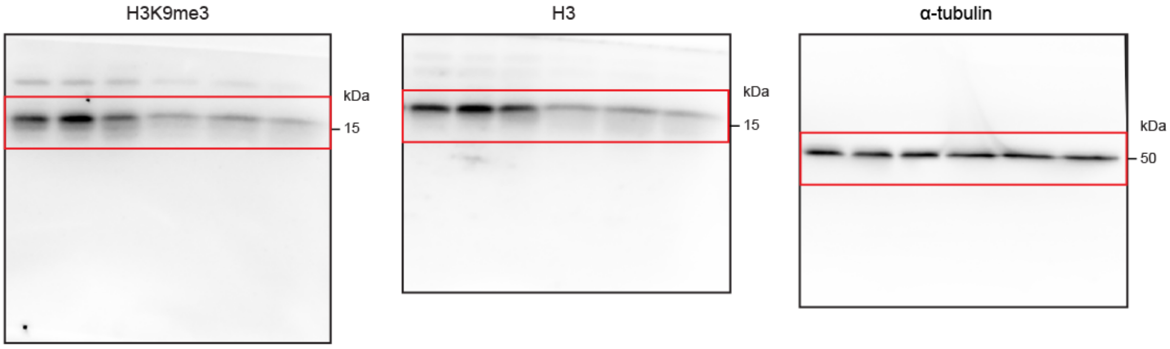

Fig. 10c

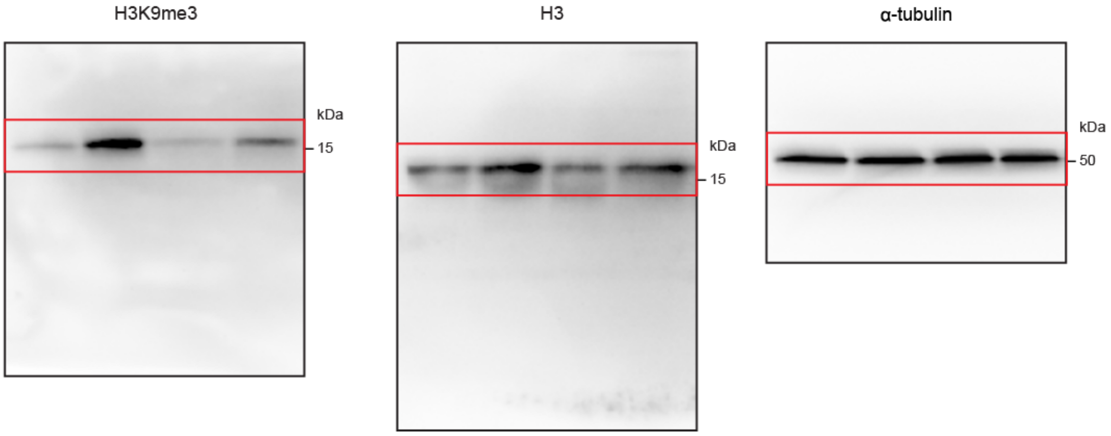

Supplement: Supplementary file 9 — Supplementary Data 7 [file 42003_2020_984_MOESM9_ESM.pdf]
