# Supplementary material for: The ubiquitin-conjugating enzyme UBE2K determines neurogenic potential through histone H3 in human embryonic stem cells
Source: Commun Biol. 2020 May 25;3:262. doi: 10.1038/s42003-020-0984-3 (PMC7248108; doi:10.1038/s42003-020-0984-3)
Supplement: Supplementary file 11 — Reporting Summary [file 42003_2020_984_MOESM11_ESM.pdf]

nature research

Corresponding author(s): David Vilchez

Last updated by author(s): Apr 21, 2020

Reporting Summary

Nature Research wishes to improve the reproducibility of the work that we publish. This form provides structure for consistency and transparency in reporting. For further information on Nature Research policies, see Authors & Referees and the Editorial Policy Checklist.

Statistics

For all statistical analyses, confirm that the following items are present in the figure legend, table legend, main text, or Methods section.

n/a	Confirmed	
☐	☒	The exact sample size (*n*) for each experimental group/condition, given as a discrete number and unit of measurement
☐	☒	A statement on whether measurements were taken from distinct samples or whether the same sample was measured repeatedly
☐	☒	The statistical test(s) used AND whether they are one- or two-sided<br>*Only common tests should be described solely by name; describe more complex techniques in the Methods section.*
☒	☐	A description of all covariates tested
☒	☐	A description of any assumptions or corrections, such as tests of normality and adjustment for multiple comparisons
☐	☒	A full description of the statistical parameters including central tendency (e.g. means) or other basic estimates (e.g. regression coefficient) AND variation (e.g. standard deviation) or associated estimates of uncertainty (e.g. confidence intervals)
☒	☐	For null hypothesis testing, the test statistic (e.g. *F*, *t*, *r*) with confidence intervals, effect sizes, degrees of freedom and *P* value noted<br>*Give P values as exact values whenever suitable.*
☒	☐	For Bayesian analysis, information on the choice of priors and Markov chain Monte Carlo settings
☒	☐	For hierarchical and complex designs, identification of the appropriate level for tests and full reporting of outcomes
☒	☐	Estimates of effect sizes (e.g. Cohen's *d*, Pearson's *r*), indicating how they were calculated

Our web collection on statistics for biologists contains articles on many of the points above.

Software and code

Policy information about availability of computer code

Data collection	No software was used
Data analysis	We used GraphPad PRISM 6 (version 6.0h) for statistical analysis. We used Image J (1.51s) to quantify densitometry of immunoblots. Proteomics data sets were analyzed with the MaxQuant software (version 1.5.3.8). We employed the LFQ mode and used MaxQuant default settings for protein identification and LFQ quantification. All downstream analyses pf proteomics data were carried out on LFQ values with Perseus (version 1.5.2.4). RNA-seq and ChIP-seq data were analysed using a QuickNGS pipeline (Wagle, P., Nikolic, M. & Frommolt, P. QuickNGS elevates Next-Generation Sequencing data analysis to a new level of automation. BMC Genomics 16, 487, (2015)). This workflow system provided basic read quality check using FastQC (version 0.10.1) and read statistics using SAMtools (version 0.1.19). The basic data processing of the QuickNGS pipeline consists of a splicing-aware alignment using Tophat2 (version 2.0.10) followed by reference-guided transcriptome reassembly with Cufflinks2 (version 2.1.1). The QuickNGS pipeline calculated read count means, fold change and P-values with DEseq2 (version 1.4.5) and gene expression for the individual samples with Cufflinks2 (version 2.1.1) as FPKMs, using in both cases genomic annotation from the Ensembl database version 87.

For manuscripts utilizing custom algorithms or software that are central to the research but not yet described in published literature, software must be made available to editors/reviewers. We strongly encourage code deposition in a community repository (e.g. GitHub). See the Nature Research guidelines for submitting code & software for further information.

Data

Policy information about availability of data

All manuscripts must include a data availability statement. This statement should provide the following information, where applicable:

- Accession codes, unique identifiers, or web links for publicly available datasets
- A list of figures that have associated raw data
- A description of any restrictions on data availability

RNA-seq and ChIP-seq data have been deposited in Gene Expression Omnibus (GEO) under the accession code GSE146704. The mass spectrometry proteomics data

showed in Supplementary Data 2 and Supplementary Figure 12 have been deposited to the ProteomeXchange Consortium via the PRIDE74 partner repository with the dataset identifiers PXD018625 and PXD018621, respectively. Uncropped images of western blots are presented in Supplementary Data 7. All source data underlying the graphs presented in the main figures can be found as Supplementary Data 8. All the other data are also available from the corresponding author upon request (DV).

Field-specific reporting

Please select the one below that is the best fit for your research. If you are not sure, read the appropriate sections before making your selection.

☒ Life sciences ☐ Behavioural & social sciences ☐ Ecological, evolutionary & environmental sciences

For a reference copy of the document with all sections, see nature.com/documents/nr-reporting-summary-flat.pdf

Life sciences study design

All studies must disclose on these points even when the disclosure is negative.

Sample size	In each independent experiment using mammalian cells, biological replicates/wells were averaged for every condition. Then, the data of different independent experiments (at least 3) were averaged. Finally, we compared this average across conditions/groups. For C. elegans experiments, sample size determination was done according to standard C. elegans approaches. Sample sizes are indicated in the corresponding figure legends and Supplementary Information.
Data exclusions	No data were excluded from the analyses.
Replication	At least three independent experiments for each assay were performed to verify the reproducibility of the findings (if there were two independent experiments, this was also noticed in the figure legend). All the attempts of replication gave a similar outcome. Exact numbers and statistics are provided in figure legends and supplementary data.
Randomization	For all experiments with mammalian cells, cells with similar confluence were splitted and equal amount of cells were transferred to new plates for experiments. The plates were randomly assigned to the different treatment conditions. The different samples and conditions were lysed and analyzed in random order.<br>For experiments with C. elegans, worms were synchronized by bleaching of young hermaphordite worms followed by L1 starvation standard procedures. These young hermaphrodite worms were obtained by transferring random chunks of agar from maintenance plates and let them grow until we have sufficient young hermaphrodites worms for bleaching. After bleaching worms and obtaining synchronized adults, these young worms were randomly assigned to the different treatment conditions. Then, the samples were collected, lysed and analyzed in random order.
Blinding	The samples and different conditions were not processed in a blinded manner by the researchers participating in this study. However, cell plates and worms were randomly assigned to the different treatment conditions, the different conditions were assessed in random order and the replicate experiments were usually performed by different authors involved in the study.

Reporting for specific materials, systems and methods

We require information from authors about some types of materials, experimental systems and methods used in many studies. Here, indicate whether each material, system or method listed is relevant to your study. If you are not sure if a list item applies to your research, read the appropriate section before selecting a response.

Materials & experimental systems

n/a	Involved in the study	
☐	☒	Antibodies
☐	☒	Eukaryotic cell lines
☒	☐	Palaeontology
☐	☒	Animals and other organisms
☒	☐	Human research participants
☒	☐	Clinical data

Methods

n/a	Involved in the study	
☐	☒	ChIP-seq
☒	☐	Flow cytometry
☒	☐	MRI-based neuroimaging

Antibodies

Antibodies used	For western blot analysis, we used:<br><br>anti-UBE2K (Cell Signaling, #8226, 1:1,000), anti-OCT4 (Stem Cell Technologies, #60093, 1:500), anti-SOX2 (Abcam, #97959, 1:1,000), anti-PAX6 (Stem Cell Technologies, #60094, 1:200), anti-nestin (Stem Cell Technologies, #60091, 1:1,000), anti-MAP2 (Sigma, #1406, 1:1,000), anti-polyubiquitinylated conjugates (Enzo, PW8805-0500, 1:1,000), anti-ubiquitin (Merck Millipore, # 05-944, clone P4D1-A11 , 1:1000), anti-H3K9me3 (Abcam, #8898, 1:1,000), anti-Histone H3 (Cell Signaling, #2650, 1:10,000), anti-H3K9me1 (Cell Signaling, #1418, 1:1,000), anti-H3K9me2 (Cell Signaling, #4658, 1:1,000), anti-H3K4me3 (Active Motif, #39916, 1:1,000), anti-H3K27me3 (Active Motif, #39155, 1:1,000), anti-H3K27ac (Active Motif, #39933, 1:1,000), anti-HTT (Cell

Signaling, #5656, 1:1,000), anti-SETDB1 (Abcam, #107225, 1:500), anti-p53 (Cell Signaling, #9282, 1:2,000), anti-Histone H1 (Merck, 05-457, 1:1,000), anti-PSMD11 (Abcam, #99413, 1:1,000), anti-ß-actin (Abcam, #8226, 1:1,000) and a-tubulin (Sigma, T6199, 1:5,000).

For Immunocytochemistry , we used:

Rabbit anti-H3K9me3 (Abcam, #8898, 1:500), Rabbit anti-PAX6 (Stem Cell Technologies, #60094, 1:300), Mouse anti-OCT4 (Stem Cell Technologies, #60093, 1:200), Mouse anti-Nestin (Stem Cell Technologies, #60091, 1:500), Rabbit anti-SOX1 (Stem Cell Technologies, #60095, 1:100), Mouse anti-MAP2 (Sigma, #1406, 1:200), Rabbit anti-H3K9me3 (Abcam, #8898, 1:500).

For co-immunoprecipitation assays, we used:

anti-histone H3 antibody (Cell Signaling, #2650)
anti-FLAG antibody (SIGMA, F7425)
anti-UBE2K antibody (Cell Signaling, #8226 1:50)

Validation	Validations of antibodies were done by the stated manufacturer's and supported by the publications indicated in the manufacturer's websites.

Eukaryotic cell lines

Policy information about cell lines

Cell line source(s)	The H9 (WA09) and H1 (WA01) hESC lines were obtained from the WiCell Research Institute. The human iPSC line (ACS-1011) and parental HFF-1 fibroblasts (SCRC-1041) were obtained from ATCC. HEK293 cells were obtained from ATCC
Authentication	We have authenticated the hESC lines in the lab by performing STR analysis.
Mycoplasma contamination	All the cell lines used in this study were tested for mycoplasma contamination at least once every three weeks. No mycoplasma contamination was detected.
Commonly misidentified lines (See ICLAC register)	None of the cell lines used in this paper are listed in the database of commonly misidentified cell lines maintained by ICLAC

Animals and other organisms

Policy information about studies involving animals; ARRIVE guidelines recommended for reporting animal research

Laboratory animals	The study involved Caenorhabditis elegans strains with the following genotypes:<br>Wild-type (N2)<br>SS104 (glp-4(bn2)I)<br>CF512 (fer-15(b26)II;fem-1(hc17)IV)<br>MT13293 (met-2(n4256)III)
Wild animals	The study did not involve wild animals.
Field-collected samples	The study did not involve samples collected from the field.
Ethics oversight	In this research, we used invertebrate C. elegans as an organismal model and no ethical approval was required. According to the “Zentrale Kommission für die Biologische Sicherheit” (ZKBS), the responsible entity inside the Bundesamt für Verbraucherschutz und Lebensmittelsicherheit to assess the risk of Genetically Modified Organisms (GMO), genetic work with C. elegans is classified as risk group 1 (biological safety level 1: S1). Accordingly, we performed work on C. elegans in a S1-laboratory. The use of GMO in Germany is regulated by the “Gentechnik-Gesetz”, and we followed the guidelines applying to S1 work with GMO (i.e., documentation of the project and of the, exact description of the creation and maintenance of the genetic modification or correct waste treatment).

Note that full information on the approval of the study protocol must also be provided in the manuscript.

ChIP-seq

Data deposition

☒ Confirm that both raw and final processed data have been deposited in a public database such as GEO.

☒ Confirm that you have deposited or provided access to graph files (e.g. BED files) for the called peaks.

Data access links<br>*May remain private before publication.*	Gene Expression Omnibus (GEO) under the accession code GSE146704
Files in database submission	PEAKS_ChIP_Ube1_Input_Ube1

Files in database submission

PEAKS_ChIP_Ube2_Input_Ube2
PEAKS_ChIP_NT2_Input_NT2
PEAKS_ChIP_NT1_Input_NT1

Genome browser session (e.g. UCSC)

No longer applicable

Methodology

Replicates

For each condition, two biological replicates from independent experiments were analysed

Sequencing depth

Libraries from H9 hESCs were sequenced with a 2 × 75bp read length on Illumina HiSeq4000

Antibodies

anti-H3K9me3 antibody ChIP Grade (Abcam, #8898, rabbit polyclonal, reported suitable for ChIP)

Peak calling parameters

For peak calling, the resulting Binary Alignment/Map (BAM) files were analyzed with MACS2 version 2.0.1062. The results comprise lists of significant peaks compared with the respective input DNA controls. QuickNGS pipeline identifies all genes which are 10,000 bp up- or downstream from the MACS2 peaks. To identify differential read-enriched peak regions from ChIP-seq data between different conditions, we used bdgdiff module of MACS2.

Data quality

Quality check of sequencing data were performed with FastQC version 0.10.1 (Babraham Bioinformatics).

Software

ChIP-seq sequencing reads were mapped with Burrows-Wheeler Aligner (BWA)61 to the Homo Sapiens genome (Ensembl database version 87). For peak calling, the resulting Binary Alignment/Map (BAM) files were analyzed with MACS2 version 2.0.1062. The results comprise lists of significant peaks compared with the respective input DNA controls. QuickNGS pipeline identifies all genes which are 10,000 bp up- or downstream from the MACS2 peaks. To identify differential read-enriched peak regions from ChIP-seq data between different conditions, we used bdgdiff module of MACS2. Data was uploaded into MySQL database. QuickNGS also provides password-protected track hubs for the UCSC Genome Browser with direct hyperlinks for visualization.
